# Drivers of Post-Harvest Aflatoxin Contamination: Evidence Gathered from Knowledge Disparities and Field Surveys of Maize Farmers in the Rift Valley Region of Kenya

**DOI:** 10.3390/toxins14090618

**Published:** 2022-09-03

**Authors:** Grace Gachara, Rashid Suleiman, Sara El Kadili, Essaid Ait Barka, Beatrice Kilima, Rachid Lahlali

**Affiliations:** 1 Department of Food Sciences and Agro-Processing, School of Engineering and Technology, Sokoine University of Agriculture, Morogoro P.O. Box 3006, Tanzania; 2 Southern Africa Centre of Excellence for Infectious Diseases (SACIDS), SACIDS Foundation for One Health, Sokoine University of Agriculture, Morogoro P.O. Box 3019, Tanzania; 3 Department of Animal Production, Ecole Nationale d’Agriculture de Meknès, Km10, Rte Haj Kaddour, BP S/40, Meknès 50001, Morocco; 4 Unité de Recherche Résistance Induite et Bio-Protection des Plantes-EA 4707, Université de Reims Champagne-Ardenne, 51100 Reims, France; 5 Phytopathology Unit, Department of Plant Protection, Ecole Nationale d’Agriculture de Meknès, Km10, Rte Haj Kaddour, BP S/40, Meknès 50001, Morocco

**Keywords:** stored maize, aflatoxins, post-harvest practices, Hybrid-6 series maize cultivars, food security, Rift Valley, Kenya

## Abstract

Maize-dependent populations in sub-Saharan Africa are continually exposed to aflatoxin poisoning owing to their regular consumption of this dietetic cereal. Being a staple in Kenyan households, consumption of maize-based meals is done almost daily, thereby exposing consumers to aflatoxicoses. This study assessed awareness levels, knowledge disparities, and perceptions regarding aflatoxin contamination at the post-harvest phase among farmers in the Rift Valley Region of Kenya. Households were randomly selected using a geographical positioning system (GPS) overlay of the agro-ecological zones within Uasin Gishu and Elgeyo Marakwet counties. Face-to-face interviews were conducted in 212 smallholder and large-scale farms. The study documented the demographic profiles of farmers and knowledge, awareness, and perceptions of aflatoxin contamination using a pre-designed structured questionnaire. Most farmers were familiar with aflatoxins and the adverse effects they present to health (61.32%). Almost all the farmers (94.37%) were aware of storage molds and food-spoilage fungi. However, few farmers adopted good post-harvest practices (PHPs), such as avoiding premature harvests (49.8%), using well-ventilated storage spaces (44.6%), grain sorting (30.5%), proper drying of maize (17.8%), and using hermetic bags for storage (30.5%). Conclusively, intensified farmer education is required to train farmers on good PHPs to protect their maize from aflatoxigenic fungi and aflatoxin accumulation.

## 1. Introduction

Tropical food systems increasingly remain predisposed to frequent mycotoxin outbreaks that cripple all possibilities of them being self-sustaining. In Kenya, massive attention is always diverted towards the localities where fatal mycotoxicoses tend to occur, while other regions, though being equally at risk, tend to be neglected. A classic example are the lethal aflatoxicosis outbreaks that have transpired in Eastern Kenya spanning over several years. All of them have received overwhelming attention [1,2,3,4,5], whilst agricultural zones in the western regions remain unexplored. The Rift Valley region of Kenya is one such location where little research on mycotoxin and aflatoxin contamination has been conducted although it is the country’s food basket, particularly when it comes to maize cultivation and production. The aforementioned geographical location produces approximately 80% of maize countrywide [6]. Consumers across Kenya rely on these maize for their self-reliance, a factor that denotes the importance of assessing the aflatoxin situation in this region.

Zero aflatoxin or mycotoxin outbreaks have been reported in the Rift Valley, and the lack of surveillance programs could be solely responsible for this observation. In a singular study, Mutegi et al. [7] reported high prevalence of aflatoxin contamination in peanuts (*Arachis hypogaea* L.), but since its consumption is not as widely popular as maize, the revelation did not receive much attention. Being a tropical country, Kenya primarily cultivates its maize under agro-climatic conditions that are known to accelerate fungal colonization and subsequent mycotoxin multiplication [8,9]. The majority of people practicing maize cultivation are resource-poor farmers whose pre- and post-harvest practices easily subject the cereal to increased mycotoxin contamination. Further, the Kenyan maize-value chain is quite porous in that the sequential process from production to consumption lacks any robust mechanisms of ensuring food safety [5], and contaminated grains can easily leak through and find their way into markets and retail outlets. Definitively, mycotoxin surveillance along this value chain would be significantly helpful in formulating mitigation strategies for this menace.

Maize is the primary staple in Kenyan households, with families relying on the crop for subsistence and commercial purposes. Most often, a typical family set-up will consume some of the maize they cultivate, sell a fraction of it to generate income, and store the reminder for future sustenance [10]. Neighborhood hammer mills (*posho mills*) are popular sites across Kenyan villages where people take their maize to be milled [8,11]. The clients typically mill maize weighing between 2 to 20 kg primarily for their own consumption over several days or weeks. Owing to the popularity of mills in most Kenyan villages, they often pose as strategic sites for mycotoxin build-up and accumulation in post-harvest milled maize. Prior to milling, there are already a plethora of factors that accelerate the vulnerability of maize kernels to mycotoxin while still in storage. Abiotic stress, genetic factors, and biotic elements cumulatively contribute to fungal colonization in stored grains [12]. Insects and pathogens account for the greatest biotic constraint, which accelerates aflatoxin accumulation and subsequent toxigenesis. All the aforementioned parameters, coupled with poor post-harvest practices and management, increasingly place maize and other cereals at elevated risk of mycotoxin contamination. Notably, the toxigenicity levels of the fungal populations is an important variable that determines the magnitude of mycotoxin contamination. For example, *Aspergillus flavus* has varying toxin-production genotypes, all of which have been reported to be recovered in Kenyan foods and cereals [4,8,13]. In concomitance with the aforementioned, maize genotypes tend to differ in their susceptibility to aflatoxin contamination, an aspect that highlights the importance of selecting superior breeding varieties for mycotoxin management and mitigation. Kenyan cultivated maize normally comprises both hybrid and openly-pollinated varieties [8]. Both breeds lack the two essential multiple genes that are known to provide resistance to mycotoxin infection [14,15]. The accumulation of aflatoxins is often determined by both fungal and maize genotypes, denoted as G_F_ and G_M_, respectively. Secondary factors, including farming (F) practices and environmental (E) parameters, also play a pivotal role in mycotoxin contamination.

The interplay between G_F_ × G_M_ × E × F and associated factors exacerbates the mycotoxin menace in cereals such as maize. Further, the characteristics of these grains, such as their kernel texture and type of endosperm, are genetic traits that predispose them to aflatoxin contamination both at the pre- and post-harvest levels. Mycotoxin analysis in Kenya (and most sub-Saharan African countries) is usually problematic due to the high costs associated with it. Consequently, few developing countries have the capacity to monitor mycotoxin amounts within their staple food systems [8]. The situation in Kenya is wanting, and despite the government having adopted the World Food Program (WFP) regulatory limits, implementation at the national level remains deficient [16,17]. Furthermore, the lack of capacity to enforce streamlined mycotoxin-monitoring systems worsens the aflatoxin problem in the country. Currently, aflatoxin limits set by the WFP stand at 10 μg/kg, but without stringent enforcement by government agencies, consumers continue to be placed at risk of eating mycotoxin-laced cereals and grains.

The objectives of this study were to assess the magnitude of aflatoxin contamination in two major maize cultivation regions where minimal research on mycotoxin prevalence has been conducted, namely Uasin Gishu and Elgeyo Marakwet counties located in the Rift Valley Region of Kenya. The study further sought to investigate the main drivers of post-harvest aflatoxin contamination by assessing knowledge disparities by conducting field surveys among both large-scale and small-scale maize farmers.

## 2. Results

### 2.1. Socio-Demographic Profile of Participants

Dominance in land ownership was observed in males, with at least more than half of the participants (62.74%) being male owners of the maize farms, and 37.26% were female owners (Table 1). The respondents were mixed smallholder and large-scale farmers, with their land sizes ranging between 1–3000 acres. Most of the farmers we interviewed (49%) halted their studies at the secondary school level, with only a few (24%) having acquired university or tertiary education. The average household size of most farmers in the study region comprised roughly five to eight family members, with majority of participants being in the age range of 30–50 years old (89%). Overall, 61% percent of respondents were well-aware of aflatoxins and their adverse health effects. In fact, 2.8% of farmers admitted that when they spotted moldy maize stovers, they would not give it to their dairy cattle, as they were aware of the aflatoxin carry-over in milk (aflatoxin B1 hydroxylation to M1).

**Figure 1:**
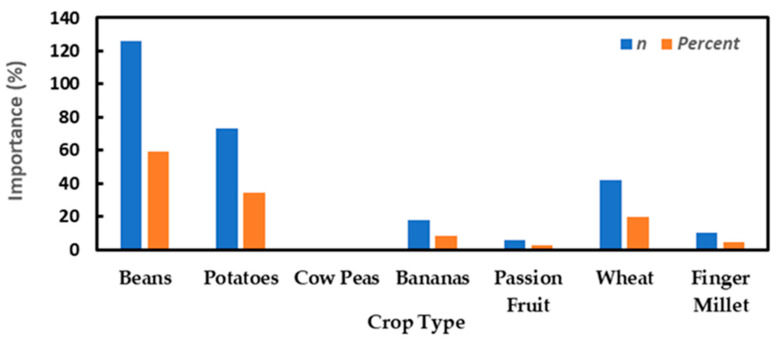
Alternative crops cultivated apart from maize through either mixed or mono-cropping systems.

Maize farming was a primary source of income for 93.9% of farmers and secondary source for 85.45% of them, implying that maize is the source of sustenance for their day-to-day living (96.7%) and commercial sale (73.7%). A lesser fraction of the respondents depended on employment (2.35%) or business (3.76%) as their primary sources of income. For farmers whose secondary activities were not tied up in farming, they admitted to having established businesses (7.98%) or taking part in different forms of craftsmanship (0.94%). Given that maize takes several months (4–6) to grow and mature, farmers explained that they opted to plant alternative crops that have shorter maturity timespans. The most commonly alternative planted crops include beans (59.15%), potatoes (34.27%), wheat (19.72%), finger millet (4.69%), passion fruit (11.02%), and bananas (8.45%) (Figure 1). Other crops also planted, albeit rarely, include sweet potatoes, sugarcane, lemon, grass, kales, spinach, oats, and pumpkins.

### 2.2. Maize Cultivation and Farming Practices of Rift Valley Farmers

**Figure 2:**
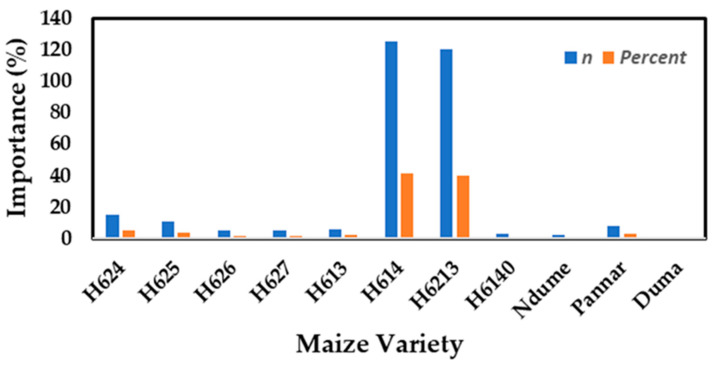
Maize varieties commonly cultivated by farmers in the Rift Valley Region.

Farmers across Uasin Gishu and Elgeyo Marakwet practice both small- and large-scale farming depending on available land acreage. Maize varieties commonly grown in the Rift Region are the hybrid series, with Hybrid-614 (H614) being the most popular among farmers at 41.53%, followed closely by H6213 at 39.87% (Figure 2). Some farmers opted for indigenous maize varieties such as Ndume, Pannar, and Duma due to their large cob size, disease tolerance, high yielding abilities (Table 2), and kernel type (Table 3). The majority of the participants were seasoned farmers, with 44.43% having practiced maize cultivation for more a period ranging ten to twenty years. Among the surveyed farmers, 40.09% admitted to being new entrants in maize farming, having cultivated the cereal crop for a period ranging from one to ten years. When it comes to sourcing seeds, most of the farmers opted to buy their preferred seed cultivars from the agrovet (46.45%), with an almost similar number of them (45.97%) purchasing it from the Kenya Seed Company, a licensed government parastatal mandated with the sale and dispensation of certified maize seeds.

A smaller fraction sourced their seeds from alternate places such as the local market, Apollo, One Acre Fund, and the Eldoret Showground. When farmers were asked about the most common diseases and infestation they faced on their crops, 93% admitted that fall armyworm infestation greatly affected their maize while still in the field. Other diseases still encountered included head smut (16%), leaf blight (5.6%), leaf rust (1.9%), and gray leaf spot (2.3%). Additionally, the farmers confessed that apart from the fall armyworm infestation, other challenges plagued the maize production process. This included the high cost of farm inputs (80.3%), expensive pricing of maize seeds, climate change (80.3%), and declining soil fertility (19.7%).

### 2.3. Post-Harvest Practices

#### 2.3.1. Grain Sorting and Grain Damage

The majority of the farmers (94.37%) admitted to sorting their grains prior to storage (Table 4). The sorting is done at different stages, with some farmers opting to sort their maize during the drying phase, while others sorted theirs while the grains were in store. The sorting criteria used by farmers involved the following: moldy maize, immature maize, insect-damaged (mainly weevils and larger grain borer) kernels, and rotten maize cobs/kernels.

#### 2.3.2. Storage Practices

**Figure 3:**
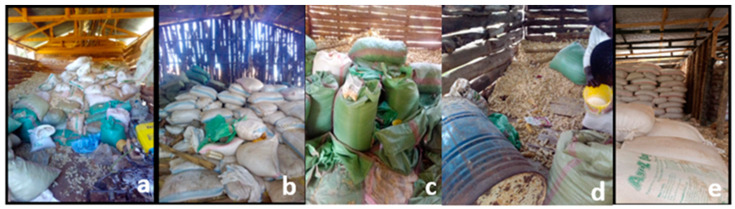
Display of how farmers in both Uasin Gishu and Elgeyo Marakwet store their maize using normal sisal bags. (**a**) Maize stored in normal sisal bags and kept on the floor; (**b**) maize stored similarly to (**a**) but in a poorly constructed granary; (**c**) maize harvest stored in poorly sealed sisal bags; (**d**) maize cobs scattered on the floor; (**e**) maize stored in well-sealed sisal bags and arranged neatly inside the granary.

**Figure 4:**
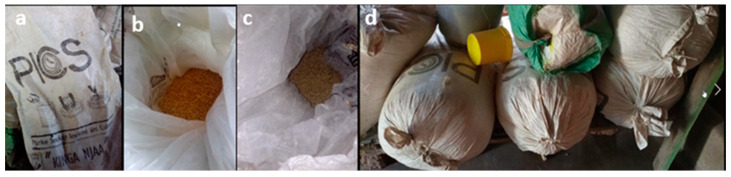
PICS bags used by Rift Valley farmers to store maize produce. (**a**) Pictorial illustration of a PIC bag with (**b**,**c**) showing maize grains stored inside; (**d**) maize stored in tightly sealed PICS bags.

**Figure 5:**
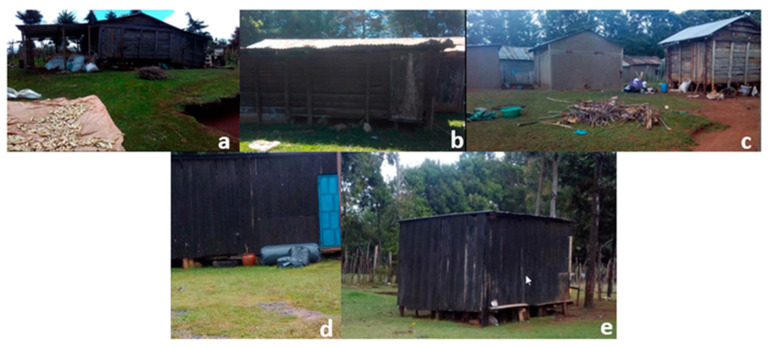
Illustration of the different storage structures within the Rift Valley. (**a**–**c**) semi-permanent wooden granaries built with minimal elevation from the ground; (**d**,**e**) semi-modern wooden granaries raised slightly from the ground to deter entry of ro-dents and crawling pests.

Across the Rift Valley, maize farmers construct their own storage facilities/structures, and they store their cereal in a somewhat unilateral fashion using sisal bags, locally termed as *gunias* (Figure 3). The type of sisal bag differs from one farm to the next and is mostly an oscillation between the one-layered sisal bag and the newly improved triple-layered Purdue improved crop-storage (PICS) bag (Figure 4). The latter, which is popularly known as the hermetic bag, is preferred for its multifaceted benefits, including minimization of insect and pest invasion, significant mold growth reduction, prolonged safe storage of cereals, and eradication of pesticide usage since the bags are already pre-treated during manufacture. Farmers who stored their maize in hermetic bags reported almost zero percent grain losses as opposed to those who used the normal sisal bags, where grain loss would sometimes reach an alarming 50%. Depending on the type of bag used, the farmers place their cereals in either the sisal or PICS bag and then put them in the store facility for long-term storage (Figure 5). In addition, 38.21% of farmers stored their maize in hermetic bags, while 50% stored theirs in the sisal bags (Figure S3). After being placed in the bag of choice, the farmers stored the maize either in the traditional granary (40.48%), modern store (21.90%), or inside their houses (37.62%). While there, the maize would be stored for a period of one month to one year, while the farmer either utilizes it for consumption or commercial purposes.

#### 2.3.3. Chi-Square Test for Association between Aflatoxin Awareness and Post-Harvest Practices

When the chi-square test was applied in testing the fit of association between knowledge of aflatoxin and the variables of gender and level of education, the former showed a significant difference (*p* < 0.05), indicating that gender plays a pivotal role in aflatoxin awareness and management in the study region. The variables of age, county of residence, income-generating activity, and level of education were not significantly associated with knowledge of aflatoxins, as their *p*-values (Table 5) were all greater than the level of significance (*p* > 0.05).

### 2.4. Farmers Knowledge and Perceptions of Aflatoxin Contamination

Most farmers (61.32%) in all the sub-counties sampled admitted to being aware of aflatoxin contamination and its harmful effects, with 38.68% being unaware of the mycotoxin (Table 6). Interestingly, 54.93% of famers had heard about aflatoxin contamination cases in their locality, while 45.07% had not heard about it.

When it comes to plant fungi and associated diseases, most farmers (96%) were aware of fungal-associated diseases, while 54.50% had no knowledge about it. Respondents adopted various post-harvest practices in striving towards minimizing aflatoxin contamination, where 17.8% dried maize adequately before storage, 30.5% sorted their maize thoroughly prior to storage, 49.8% avoided premature harvests, while 30.5% used hermetic bags for storage, and lastly, 8.5% applied pesticides before storage. Majority of the farmers (95.3%) expressed their interest in adopting good agricultural practices (GAP) to mitigate and prevent aflatoxins and aflatoxigenic fungi from contaminating their crops. Moreover, 71.4% endeavored to embrace quality post-harvest practices, which are critical in the prevention of aflatoxin accumulation in maize grains. For this instance, 76.1% and 75.1% of farmers explained they would use proper storage bags and improve their storage facilities, respectively.

## 3. Discussion

The present study sought to investigate the knowledge disparities, perceptions, and awareness levels of aflatoxin contamination among maize farmers residing in the Rift Valley Region of Kenya. Recurrent outbreaks of acute aflatoxin poisoning and fatal aflatoxicosis in Kenya associated with consumption of contaminated maize are often reported in the Eastern Region (Machakos, Makueni, and Kitui). Hardly do these reports highlight any outbreaks in the Western or Rift Valley Regions, which could equally be possible risk-alert areas. It remains unsubstantiated whether mycotoxins are actually a periodic, sporadic, or chronic problem in the aforementioned areas where these fatalities have not yet been reported. Deemed the breadbasket of Kenya, the Rift Valley produces the bulk of Kenyan maize and is primarily where the cultivation and production of this important cereal is done majorly in large scale. With agriculture generating revenue and income for more than half of the households residing in Uasin Gishu and Elgeyo Marakwet Counties, the importance of farming in this area cannot be overemphasized.

In the current study, we endeavored to extend the understanding of the aflatoxin situation in the Rift Valley, Kenya’s highest maize-producing region, through increased interviewing of farmers whilst undertaking farm assessments across multiple locations. The regional survey specifically targeted the post-harvest level, particularly storage, while [7] comparing the findings to pre-harvest parameters such as climatic patterns, cropping systems (mono-cropping versus mixed cropping), harvesting techniques, and other important farm-management practices. Approximately 78% of people residing in Uasin Gishu and Elgeyo Marakwet Counties earn their living primarily through engagement in crop farming and livestock husbandry [18]. Nonetheless, despite these regions being the trailblazers in maize farming, scarce comprehensive mycotoxin surveys have been conducted in the region to ascertain whether there is any prevalence of aflatoxins. Scanty literature exists on the occurrence and distribution of aflatoxins in maize in Uasin Gishu and Elgeyo Marakwet counties, with the existent articles shedding minimal insights on the prevailing situation. The few available publications are solely based on data collected from either small geographical zones or small sample sizes [19,20].

Our study revealed that even though a fraction of the farmers were well-versed with aflatoxin contamination at post-harvest, most of them still required intensive training to be taught about the importance of adhering to good post-harvest practices and how these would protect their maize from aflatoxin accumulation. For the farmers who were well-versed with aflatoxigenic fungi, they went ahead to opt for the hermetic and PICS bags for storing their maize instead of the normal sisal bags. Apart from protecting their maize from invasion by *Aspergilli* molds, the farmers reiterated that these bags equally cushioned their cereal from infestation by storage pests such as weevils and grain borers. However, the usage of PICS bags amongst large-scale farmers was only limited to the maize intended for household consumption, whilst the maize meant for sale was stored in normal sisal bags. Farmers cited the high cost of hermetic bags as the primary reason for opting to store maize intended for commercial distribution in the usual *gunias.* Evidently, it becomes apparent that consumers purchasing maize from such farmers are placed at a higher predisposition of feeding on maize possibly contaminated by aflatoxins given that some of the farmers would store their maize for over one year or until the period all the cereal in store has been sold. Research has shown that the longer maize is kept in storage, the higher the likelihood of the proliferation and multiplication of aflatoxigenic fungi. The situation is even made direr for farmers who do not use the hermetic bags at all for storage and instead opt for the sisal bags.

Given that most maize farmers in the Rift Valley store maize in traditional and semi-modern structures (Figure 3, Figure 4 and Figure 5), which sometimes have large crevices and leaking roofs, the multiplication rate of these aflatoxin-producing fungi is further accelerated. From this illustration, it becomes evident that aflatoxin contamination in Rift Valley maize could be a possible occurrence given all the aforementioned scenarios. Farmers in Uasin Gishu particularly mentioned that sometimes, their maize would overstay in storage (>9 months) during the seasons when market prices were not too favorable. Therefore, instead of incurring losses, they would opt to hoard their maize until the time when prices would favor their produce. Consequently, farmers unknowingly expose their maize to greater risk of invasion of aflatoxigenic molds since prolonged storage favors fungal multiplication. In previous seasons, farmers in the Rift Region reported that their maize went moldy in store resulting in their loss of income and source of food in a two-fold fashion. Given that the interviewed farmers primarily plant maize for subsistence use and commercial sale (96.7% and 73.7%), respectively, it is then deemed paramount to evaluate the exact measure of risk of aflatoxin contamination in this maize that ideally feeds the larger part of the Kenyan population. Previous studies have shown that purchased maize contained higher aflatoxin levels compared to home-grown maize. Mutiga et al. [8] cited that Kenyans valued maize they cultivated and harvested themselves more than what they purchased either in local millers or markets.

Hoffmann and Gatobu [21] concurred with this observation, reiterating that people value home-grown maize more because they are sure of its safety and quality. The current study illustrates the truth in this observation where Rift Valley farmers cushioned themselves against aflatoxin contamination and grain losses by storing maize meant for household consumption in hermetic bags and that meant for sale in the ordinary sisal bags. The threat of aflatoxin accumulation in Rift Valley maize is not only brought about by poor post-harvest practices but also the climate change menace. The latter phenomenon affects maize farming both at the pre-harvest and post-harvest level. In this study, certain geo-climatic locations exhibited a higher predisposition for aflatoxin accumulation (Table S1) owing to the shifting oscillations in temperature and annual precipitation. In studies conducted by Mutiga et al. [8] and Yard et al. [22], aflatoxin exposure was reported to be widespread across Kenya at a time when no outbreak cases had been reported or previously flagged. These studies implied that contamination of foods by aflatoxin is actually a common occurrence in staples such as maize but often goes unnoticed due to lack of national surveillance programs. The findings by Yard et al. [22] demonstrated a dire need for mycotoxin-monitoring programs countrywide, even in locations flagged off as low-risk aflatoxin-contamination regions.

The survey also observed that mono-cropped maize had higher predisposition of being exposed to aflatoxin contamination compared to intercropped or mixed-cropped maize. The latter findings concur with those of Mutiga et al. [23], who observed the same in a longitudinal survey in Eastern Kenya. However, the reports contradict previous findings of Hell [24] and Tédihou et al. [25], who carried out studies to investigate the correlation between intercropping and aflatoxin levels in maize. In their study, both aforementioned authors showed that there was no significant reduction in aflatoxin levels even if farmers practiced intercropping. Often, the latter is adopted by most farmers with the primary objective of increasing the diversity and nutritional content of their food crops. For instance, legumes will be intercropped with cereals to aid in boosting soil nitrogen. Additionally, intercropping has been shown to reduce plant stress by suppressing weed germination, minimizing rates of evapotranspiration, and even eradicating the occurrence of certain diseases [26,27]. Given the success of this approach, it would be interesting to investigate if there are any specific crops that can be intercropped with maize to resultantly reduce aflatoxin accumulation.

Previous research has shown that age is an important parameter to consider in farming, and according to Adesina and Zinnah [28] and Adesina et al. [29], older farmers are often more likely to embrace new innovations, especially those associated with the elimination of recurrent or perennial problems such as aflatoxins. Furthermore, education is an instrumental determinant of technological acceptance and adoption because it normally tends to minimize the likelihood of risk aversion for farmers, thereby enabling them to try out new innovations [29]. According to the data generated, 49.30% of the farmers interviewed had attained secondary education or its equivalent. In general, their education level grants them higher advantage to embrace modern inventions as opposed to their uneducated counterparts. It could also be argued that poverty is both a consequence and cause of low adoption of technology. In itself, poverty amplifies the aversion of risk, especially among impoverished households. The latter are more likely to forego profitable or beneficial technologies, which, despite being risk-laden, have the ability to significantly improve their crop yields and subsequently their income altogether. Dercon and Christiaensen [30] stated that poor households are focused more on avoiding losses and hence will easily brush of newer technologies even if they can be of greater advantage to them.

During the questionnaire-filling exercise, some farmers (2.8%) admitted that when they spotted moldy maize stovers, they would not give it to their dairy cattle, as they are aware of the aflatoxin carry-over in milk (aflatoxin B1 hydroxylation to M1). The sorting criteria used by farmers involved the following: moldy maize, immature maize, insect-damaged (mainly weevils and larger grain borer) kernels, and rotten maize cobs. According to Phokane et al. [31], sorting of maize is critical in the reduction of aflatoxin accumulation and is capable of minimizing contamination by 50%. Insects act as vectors of fungal pathogens, whereby they transfer fungal spores from the atmosphere onto maize cobs or kernels. Stathers and Mvumi [32] advised that grain damage can be drastically minimized by upholding good agricultural practices during pre- and post-harvest stages, such as threshing and handling and appropriate storage. The aflatoxin challenge in the Rift Region is additionally being worsened by the fall armyworm infestation. The fall armyworm (*Spodoptera frugiperda*) has excessively exacerbated the aflatoxin menace in the Rift Valley, where it ravages maize crops during the dry season and feeds on the succulent parts of the stems and leaves, thereby reducing maize yields by between 0.77 to 1.0 ton per hectare [33].

The fall armyworm (FAW) problem is compounded by the pest’s resistance to pesticides coupled with its rapid multiplication and reproduction capacity. Additionally, the onset of the rainy season somewhat favors the FAW in that the efficacy of chemical treatments drastically reduces when it is raining. The rainwater dilutes the active ingredient of pesticides and chemical operations, allowing FAW eggs and larva to survive and re-evolve once the rainy season is over. Within the Rift Valley, the commonly used pesticides used to fight the FAW are nimbecidine, duduthrin (lambda-cyhalothrin), and agrisil [33]. The former acts as a great repellant by preventing feeding and oviposition, while the latter two work by toughening the tissues of maize plants such that they become unattractive and less succulent for larvae. Other less common pesticides used to fight the FAW include Tremor^®^ GR 0.05, Ranger^®^ 480EC, Green Life^®^, Indoking 300SC, Nurelle D 505, and Escort^®^ 19EC. These range of pesticides work by either systemic or contact mechanism depending on the absorption rate of the available biofactors. For local farmers who are unable to afford the aforementioned pesticides, they formulate a concoction of washing powder, wood ash, tobacco extracts, and water to combat the armyworm.

The mixture is filtered, after which the filtrate is sprayed on the infested part of the maize leaf or the entire leaf whorl [34]. Candidly, the majority of the subsistence farmers in sub-Saharan Africa hardly apply pesticides to maize to control pests and instead prefer to opt for cultural control mechanisms, such as those aforementioned. Interestingly, intercropping and mixed cropping have been cited as excellent cultural control methods that either deter pests or kill them altogether. Interestingly, the Rift Valley Region has been flagged for increasing cases of esophageal cancer, a chronic condition that is correlated with consumption of mycotoxin-contaminated meals [35,36]. Recent statistics on global age-standardized cancer fatality rates have shown that Kenya ranks 8th and 76th for esophageal and liver cancers, respectively [37]. Previous co-exposure studies have shown that combined ingestion of mycotoxins (e.g., aflatoxin and fumonisin) can increase the risk of liver cancer compared to ingestion of aflatoxin alone [38,39]. Mycotoxin co-occurrence has been shown to catalyze the onset of human morbidity and stunted growth in children [40,41].

In order to prevent storage and post-harvest losses, Kenyan farmers normally apply various types of preservatives to their maize before storage. The preservatives commonly include synthetic pesticides and traditionally prepared preservatives such as ash and botanicals (plant-based derivatives). Interestingly, application of these pesticides is known to decrease mycotoxin contamination but still leaves maize predisposed to weevil damage [42,43]. In Uasin Gishu, most inhabitants are large-scale farmers with farms stretching up to 10,000 acres. Therefore, they obviously earn higher income from maize cultivation and are better placed to adopt superior post-harvest practices and maize-preservation techniques in comparison with small-scale farmers elsewhere in the country. Kaaya et al. [44] corroborated this observation with their reports of a significant correlation between aflatoxin levels and weevil damage in post-harvest maize in Uganda. Similarly, the findings by Ono et al. [45] showed that there was a positive correlation between aflatoxin presence and weevil infestation. Aflatoxin surveillance and mycotoxin monitoring is evidently paramount not only in the known hot-spot regions but also in the breadbaskets of Kenya, where there is high maize production.

## 4. Conclusions

Agriculture is the mainstay in the Rift Valley Region, particularly in Uasin Gishu and Elgeyo Marakwet Counties, where it contributes to approximately 80% of food security and household income. The concluded regional survey of maize farmers in the Rift Region showed that aflatoxin contamination is a possible risk, an aspect that requires intensified surveillance given the high maize production in both counties. Cropping systems, climate change, and post-harvest practices all have an interconnected role in the contamination of maize by aflatoxins, as evidenced by previous research findings. The detailed survey conducted on storage structures provided valuable insights on the varietal differences that may be present when it comes to aflatoxin contamination at post-harvest and in storage. Inferior and poorly constructed storage sheds, granaries, or grain barns are potential invasion points for aflatoxin accumulation. The need to adopt superior post-harvest practices is paramount, and these techniques can be augmented by national surveillance programs, educative courses to create aflatoxin awareness, and the adoption of push-and-pull farming systems. Mycotoxin-related work, specifically aflatoxin surveillance, should be designed to cover all maize farming and producing regions in Kenya and not only previously documented areas with acute aflatoxicosis outbreaks. Locations with similar agro-climatic patterns to those in the Rift Region should additionally be included in the mycotoxin-monitoring programs to alleviate all possibilities of having an unprecedented aflatoxicosis outbreak within the food baskets of Kenya.

## 5. Materials and Methods

### 5.1. Study Regions

Regional site surveys were conducted in Uasin Gishu (Figure S1) and Elgeyo Marakwet (Figure S2) counties between June and November 2021. The latter timeline allowed the survey exercise to cover both the hot and dry season together with the wet and rainy season. Both counties fall within the Rift Valley, an administrative region popularly known for large-scale cereal cultivation and production, including maize, millet, sorghum, and wheat. By far, maize accounts for the widely cultivated cereal, with nearly most farmers growing the crop in either small or large scale. Uasin Gishu covers an area of 3345 km^2^, with arable land covering 2995 km^2^, representing approximately 90% of the total county land area. The remaining 10% land cover is occupied by forestland (both plantations and indigenous) and non-arable areas such as hilly and rocky terrain. The county lies between latitudes 0.5528° N and longitudes 35.3027° E, with its estimated population standing at 1,163,186 inhabitants [46].

Elgeyo Marakwet borders Uasin Gishu to the south and covers an area of 3049.7 km^2^, with an estimated population of 454,480, and lies between latitudes 1.0498° N and 35.4782° E [46,47]. Its climatic patterns somewhat differ from those of Uasin Gishu, with the county being popularly known to have an elevated altitude, a factor that makes the region suitable for profitable mixed farming (Table S1). The corresponding agro-ecological zones (AEZ) for both counties were categorized into either of the following: (1) upper highlands (UH); (2) upper midlands (UM); (3) lower midlands (LM); (4) highlands; (5) lowlands; and (6) escarpment. Within each county, sub-counties or smaller administrative districts were selected as preferential field survey hubs. In each sub-county, villages were purposively selected and a total of 213 farmers interviewed subject to their consent to take part in the study.

### 5.2. Questionnaire Design, Development, Administration, and Data Collection

Structured questionnaires designed using KoboCollect Toolkit open-source Software (KoBoCollect v2021 1.3.4, Harvard University, Cambridge, MA, USA) were administered to maize farmers for purposes of obtaining quantitative data on post-harvest practices. The questionnaires were organized according to the following sub-sections: (1) socio-demographic information; (2) maize cultivation practices; (3) major post-harvest pests and diseases; and most importantly, (4) participant knowledge and awareness of mycotoxins. Supplementary information sought to learn more about the cropping systems, agronomic activities, various techniques used to judge the dryness of maize before storage, and methods embraced in cleaning their storage structures before loading new stock after fresh harvests. The majority of the questions were targeted to collect information that converged towards the degree of aflatoxin contamination in stored maize. Both open-ended and closed-ended questions were included in order to guarantee adequacy of the questionnaire.

Aspects related to drying of maize, storage containers, storage structures, and use (or lack thereof) of pesticides are all-important parameters when it comes to understanding the scope of aflatoxin accumulation in stored maize grains. The questionnaires were initially prepared in English language, but during administration, they were translated to local languages (Kiswahili and Kalenjin) for easier comprehension and evaluation. The questionnaires were administered to participants who fulfilled the following two-tiered inclusive criteria: were farmers of local maize cultivars and had the capacity to store maize for a period not less than 3–9 months after harvest. Centrally trained and locally hired enumerators then administered these questionnaires to selected farmers through face-to-face interviews in Uasin Gishu and Elgeyo Marakwet, respectively.

### 5.3. Statistical and Data Analysis

Field survey data were captured online using the KoboCollect Toolkit, from which it was exported to Microsoft Excel for data cleaning. For each question, the number (and percentage) of farmers who provided similar responses was calculated similarly, while in cases where farmers failed to respond to certain questions, those blanks were excluded from the calculations. In instances where the farmers cited more than one reason/response to a question, the calculations were done similarly for each group of similar responses. The e-questionnaire data was analyzed using R statistical software. Descriptive statistics (primarily frequencies and means) were computed to characterize the socio-demographic profile and attributes of farmers while also gauging their perceptions on aflatoxin contamination at the post-harvest level.

Comparative statistical tools such as chi-square and one-way analysis of variance (ANOVA) were employed in the analysis, where ANOVA was used in assessing the differences in farming practices, knowledge, and perceptions of farmers concerning aflatoxin contamination. The chi-square test was applied in determining the fit of association of knowledge of aflatoxin and the variables of gender, age, income-generating activity, and level of education. The level of significance was accepted as *p* < 0.05 at 95% confidence level.

## Figures and Tables

**Table 1:** Socio-demographic Profile of Interviewed Participants.

Parameter	Variable	*N*	%
Gender	Male	133	62.74
	Female	79	37.26
Age	20–30	8	3.79
	30–40	44	20.85
	40–50	91	43.13
	50–60	53	25.12
	60–70	11	5.21
	>70	5	1.90
Education	Informal	0	0
	Primary	55	25.82
	Secondary	105	49.30
	College/University	53	24.88
	Other	0	0
Size of Household	1–5	88	41.31
	6–10	118	55.86
	11–23	6	2.76
Primary Source of Income	Farming	200	93.90
	Employment	5	2.35
	Business	8	3.76
	Apprenticeship	0	0
	Others	0	0
Secondary Source of Income	Farming	182	85.45
	Employment	12	5.63
	Business	17	7.98
	Craftsmanship (“*juacali*”)	2	0.94
	Others	0	0
Aflatoxin Awareness	Yes	132	62
	No	81	38

*N*: Total number of participants per each socio-demographic characteristic considered. %: Percentage of participants per each socio-demographic characteristic considered.

**Table 2:** Maize Cultivation and Pre-harvest Practices of Rift Valley farmers.

Farming Parameter	Variable	*n*	%
Agro-ecological zone (AEZ)	Escarpment	36	16.90
	Highlands	10	4.69
	Lower Midlands (LM)	40	18.78
	Upper Highlands (UH)	97	45.54
	Upper Midlands (UM)	30	14.08
Land acreage under maize cultivation	1–5	138	64.79
	5–10	36	16.90
	10–20	25	11.74
	20–30	3	1.41
	30–50	8	3.76
	>50	3	1.41
Years maize farming has been practiced	1−10	85	40.09
	10–20	94	44.34
	20–30	25	11.79
	>30	8	3.77
Maize varieties cultivated	H624	15	4.98
	H625	11	3.65
	H626	5	1.66
	H627	5	1.66
	H613	6	1.99
	H614	125	41.53
	H6213	120	39.87
	Ndume *	2	0.66
	Pannar *	8	2.66
	Duma *	1	0.33
Source of maize seeds	Own	2	0.95
	Agrovet	98	46.95
	Kenya Seed Company	97	45.97
	Local Market	3	1.42
	Neighbor	1	0.47
	Others ^a^	10	4.74

**^a^** This category includes farmers who sourced their seeds from alternative outlets such as Apollo, One Acre Fund, and Eldoret Racecourse Showground. * Indigenous maize varieties locally available in Kenya and preferred by Rift Valley farmers due to their large cob size, disease tolerance, and high yielding abilities.

**Table 3:** Maize Varietal Types vs. Kernel Type: Reasons for Farmers’ Preference of Sampled Popular Maize Varieties in the Rift Valley Region of Kenya.

Maize Variety									
	H6213	H614	H624	H625	H626	H627	*Pannar*	*Duma*	*Ndume*
Parameter									
Varietal Type	Hybrid-6 Series	Hybrid-6 Series	Hybrid-6 Series	Hybrid-6 Series	Hybrid-6 Series	Hybrid-6 Series	Open-Pollinated	Open-Pollinated	Open-Pollinated
Kernel Type	Flint	Semi-flint	Semi-flint	Flint	Flint	Semi-flint	Dent	Dent	Dent
No. of Farmers (*n*)	120	125	15	11	5	5	8	1	2
Percentage of Farmers (*%*)	39.87	41.53	4.98	3.65	1.66	1.66	2.66	0.33	0.66
Cumulative Percentage of Farmers from All Categories									
Source of Seeds									
Kenya Seed Company				45.97					
Local Agrovet				46.95					
One Acre Fund				2.92					
Local Market				1.42					
Other ^a^				4.74					
Reasons Given for Variety Preference									
Higher yield quantity									
Early maturity									
Weighs heavier than other varieties									
Pest tolerance									
Rot tolerance (while in the field during the rainy season)									
Drought tolerance									
Flour quality									
Cheaper cost of seeds									

^a^ Other sources of seeds.

**Table 4:** Post-harvest practices and challenges faced by maize farmers in the Rift Valley Region.

Farming Parameter	Variable	*n*	%
Common maize pests	Gray leaf spot	5	2.3
	Common rust	4	1.9
	Maize lethal necrosis	5	2.3
	Lepidopteran maize stem borers	9	4.2
	Leaf blight	12	5.6
	Fall armyworm (FAW)	178	83.6
Methods of maize storage	On the floor	2	0.94
	Polypropylene bag	18	8.49
	PICS bag/hermetic bags	81	38.21
	Reed/sisal basket	1	0.47
	Sisal bags (“*gunias*”)	106	50.00
	Others ^b^	4	1.89
Do you sort your grains to remove visibly moldy, damaged, or degraded maize kernels	Yes	201	94.37
	No	12	5.63
Maize storage area	In the house	79	37.6
	Modern store	46	21.90
	Traditional granary	85	40.48
Length of maize storage before consumption	1–3 months	27	12.80
	3–6 months	24	11.37
	6–9 months	129	61.14
	>9 months	31	14.69
Challenges in maize production	Reduced soil fertility	42	19.7
	Increased cost of maize seeds	77	36.1
	Increased cost of farm inputs	17	8.0
	Maize diseases	14	6.6
	Climate change	63	29.6
Practices adopted in reducing aflatoxin contamination	Proper drying of maize	38	17.8
	Sorting maize during drying	65	30.5
	Store in ventilated place	35	16.4
	Using hermetic bags	65	30.5
	Applying herbicides before storage	10	8.5

**^b^** Alternative methods of storing maize included the use of plastic containers, large drums, and reed baskets.

**Table 5:** Chi-square Test for Association between Knowledge of Aflatoxin and Farmers’ Socio-demographics.

	Knowledge of Aflatoxin			
	No	Yes	Chi-square value	*p*-value
**Gender**			5.18	0.023
Male	43	89		
Female	39	40		
**Age**			1.72	0.89
>20	2	6		
>30	16	28		
>40	39	52		
>50	19	33		
>60	4	7		
>70	2	2		
**County**			1.63	0.20
Elgeyo Marakwet	29	34		
Uasin Gishu	53	96		
**Education Level**			5.55	0.062
College/University	14	38		
Primary	27	28		
Secondary	41	64		
**Income-Generating Activity**			1.09	0.58
Business	2	5		
Employment	1	4		
Farming	79	121		

**Table 6:** Knowledge, Perceptions, and Practices of Farmers Regarding Aflatoxin Contamination.

Aspect Under Investigation	Response	*n*	%
Heard about aflatoxin	Yes	130	61.32
	No	82	38.68
Heard of fungal diseases	Yes	96	45.50
	No	115	54.50
Heard of aflatoxin contamination cases in your area?	Yes	117	54.93
	No	96	45.07
Post-harvest practices (PHPs) adopted to reduce aflatoxin contamination	Proper drying of maize	38	17.8
	Sorting of grains	65	30.5
	Avoiding premature harvest	106	49.8
	Storing maize in well-ventilated places	95	44.6
	Using hermetic/PICS bags	65	30.5
	Applying pesticides before storage	18	8.5
Future plans of improving PHPs to avert aflatoxin contamination	Drying maize adequately after harvest	78	36.6
	Using proper storage bags	162	76.1
	Adopting good storage facilities	160	75.1
	Adhering to good PHPs	152	71.4
	Adopting good agricultural practices (GAP)	203	95.3

## Data Availability

All datasets are within the manuscript.

## Supplementary Materials

The following supporting information can be downloaded at: https://www.mdpi.com/article/10.3390/toxins14090618/s1: Figure S1: Map of Uasin Gishu County showing regions where field surveys were conducted; Figure S2: Map of Elgeyo Marakwet County showing regions where field surveys were conducted; Figure S3: Different storage bags used to store maize by farmers; Table S1: Geo-climatic locations, sites (farms), and bimodal rainfall patterns of regions surveyed in the Rift Valley during a regional survey in 2021. Data adapted from Ralph et al. [47].
