# Supplementary material for: Drivers of Post-Harvest Aflatoxin Contamination: Evidence Gathered from Knowledge Disparities and Field Surveys of Maize Farmers in the Rift Valley Region of Kenya
Source: Toxins (Basel). 2022 Sep 3;14(9):618. doi: 10.3390/toxins14090618 (PMC9500662; doi:10.3390/toxins14090618)
Supplement: Supplementary file 1 [file toxins-14-00618-s001.zip › toxins-1868094-supplementary.pdf]

# Drivers of Post-Harvest Aflatoxin Contamination: Evidence Gathered from Knowledge Disparities and Field Surveys of Maize Farmers in the Rift Valley Region of Kenya

**Grace Gachara, Rashid Suleiman, Sara El Kadili, Essaid Ait Barka, Beatrice Kilima, and Rachid Lahlali**

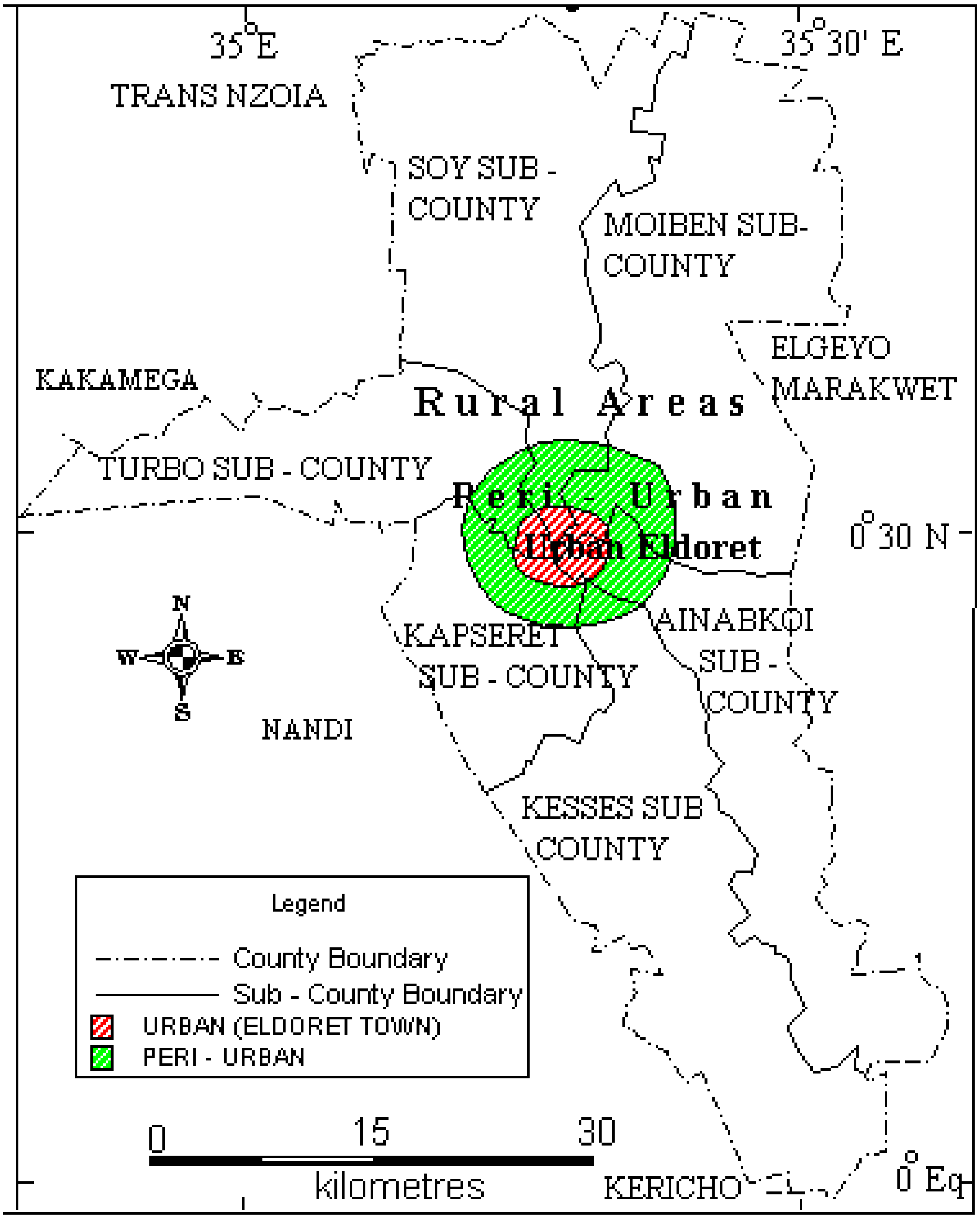


**Figure S1.** Map of Uasin Gishu County showing regions where field surveys were conducted.

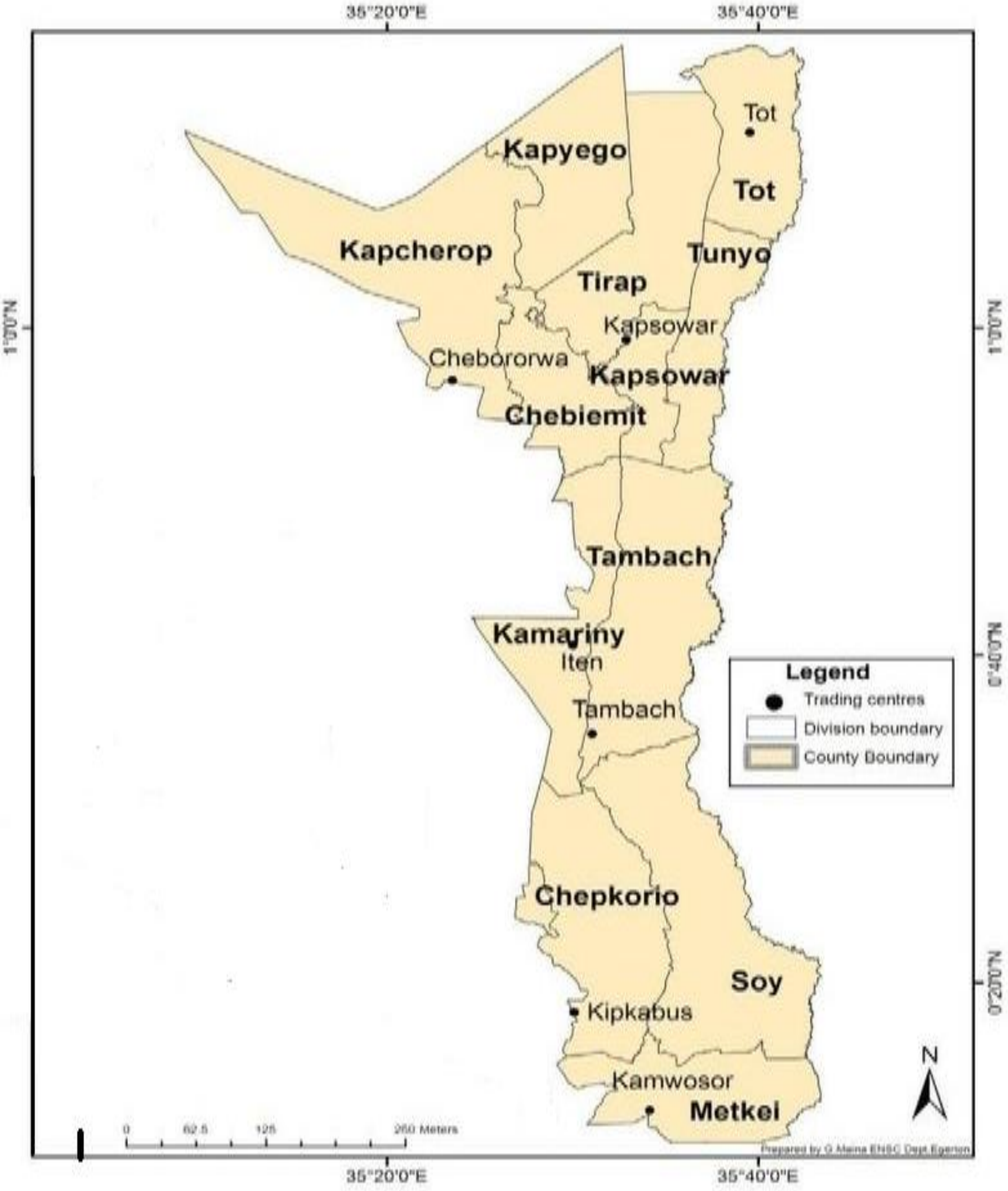


**Figure S2.** Map of Elgeyo Marakwet County showing regions where field surveys were conducted.

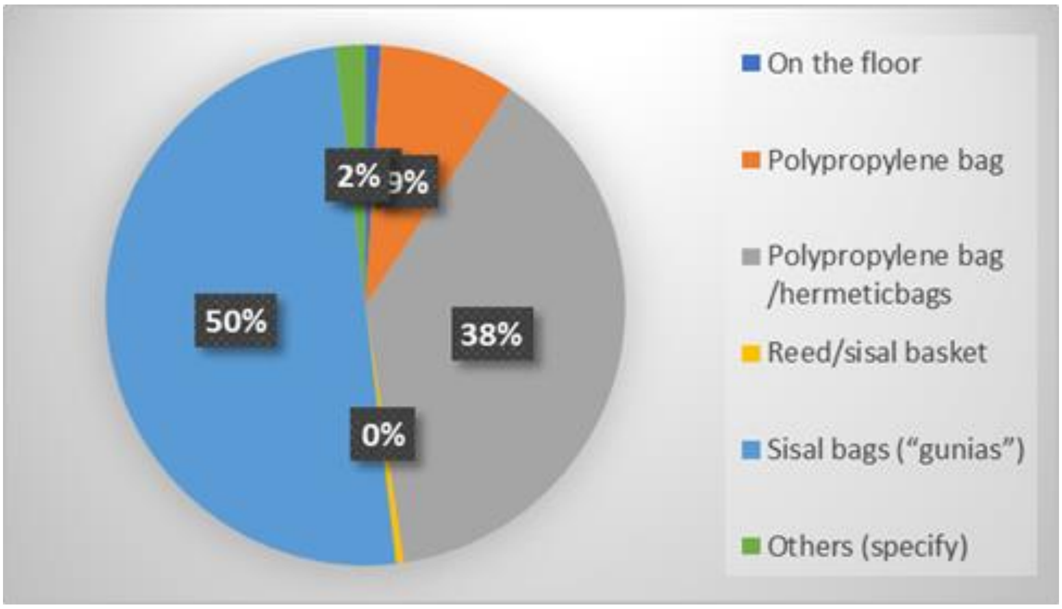


**Figure S3**. Different storage bags used to store maize by farmers

**Table S1.** Geo-climatic locations, sites (farms) and bimodal rainfall patterns of regions surveyed in the Rift-valley during a regional survey in 2021. Data adapted from Ralph [47].

| County | Sub-County (Administrative District) and Sampling Hub | Agro-ecological Zone (AEZ) Belt (Range in m) | Altitude (asl) [c] | Average Annual Temperature (°C) | Humidity[d] (%) | Rainfall Pattern [e] | Expected Rainfall Range (mm) | Received Rainfall (mm) in 2020/2021[f] |
|---|---|---|---|---|---|---|---|---|
| **Uasin Gishu** | **Eldoret**[a] | **LH3**[i] **:** 2020 –2480<br>**LH 3**[ii] **:** 1950 – 2410 | 2084-2134 | 17.3 | 72 | Unimodal | 900-1,300 | 2027 |
| | **Kapseret** | **LH3:** 1129-1562 | 900-1300 | 15.7 | 73 | Bimodal | 900-1100 | 1918 |
| | **Kesses** | **UH2:** 1913- 2119 | 2350-2450 | 16.1 | 73 | Bimodal | 1950-2450 | 1742 |
| | **Turbo** | **LH3:** 1985 – 2445<br>**UM3:** 1450 – 1910 | 1840-1859 | 18.3 | 69 | Bimodal | 900-1100 | 1372 |
| | **Soy** | **LH3:** 2041- 2239 | 1950-2450 | 15.3 | 63 | Bimodal | 900-1300 | 1527 |
| | **Ainabkoi** | LH2, LH 3 and UH4 | 2350-2450 | 15.2 | 71 | Bimodal | 1100-1200 | 1873 |
| | **Moiben** | LH2, LH 3 and UH4 | 900-1300 | 16.4 | 61 | Bimodal | 1200-1900 | 1469 |
| **Elgeyo Marakwet** | **Iten**[b] | Highlands | 1200 | 18.8 | 70 | Bimodal | 850-1000 | 1469 |
| | **Keiyo North** | Lowlands/Escarpment | 900 | 22.1 | 71 | Unimodal | 850-1000 | 1749 |
| | **Keiyo South** | Lowlands/Escarpment | 900 | 19.6 | 67 | Bimodal | 1200-2000 | 1225 |
| | **Marakwet East** | Highlands | 1500 | 21.8 | 75 | Unimodal | 1200-2000 | 1927 |
| | **Marakwet West** | Highlands | 2800 | 20.6 | 69 | Bimodal | 1300-1800 | 1749 |

[a] commercial hub and administrative center of Uasin Gishu county
[b] commercial hub and administrative center of Elgeyo Marakwet county
[c] Above sea level
[d] Humidity data retrieved from https://weather.com/
[e] cropping seasons per year which can either be bimodal (two rainy seasons) or unimodal (one rainy season)
[f] Rainfall data for the year 2020/2021 was retrieved from https://en.climate-data.org/
[i] First range of altitude in the first AEZ Belt
[ii] Second range of altitude in the second AEZ Belt
UH- Upper Highlands
LH- Lower Highlands
UM-Upper Midlands

## Reference

1. Ralph, J.; Helmut, S.; Berthold, H. *Shisanya Chris Farm Management Handbook of Kenya Vol. II-Natural Conditions and Farm Management Information,* 2nd Ed.; Part A Western Province; Ministry of Agriculture: Nairobi, Kenya, 2005; Volume II, pp. 1–221.
